# Decannulation criteria in patients with acquired brain injury based on interval forced vital capacity monitoring: a prospective observational study

**DOI:** 10.3389/fneur.2026.1798877

**Published:** 2026-07-09

**Authors:** Yunhong Deng, Fuqiang Wang, Yaojiang Li, Jinfeng Liang, Hanbo Chen, Wenying Xie, Bizhou Fu, Man Hao

**Affiliations:** 1 Clinical College of Acupuncture and Rehabilitation, Guangzhou University of Chinese Medicine, Guangzhou, Guangdong, China; 2 Department of Rehabilitation Therapy, Guangdong Sanjiu Brain Hospital, Guangzhou, Guangdong, China; 3 Department of Rehabilitation Medicine, Guangdong Sanjiu Brain Hospital, Guangzhou, Guangdong, China

**Keywords:** acquired brain injury, decannulation, forced vital capacity, pulmonary rehabilitation, threshold effect, tracheostomy

## Abstract

**Objective:**

Optimized decannulation timing in tracheostomized patients with acquired brain injury (ABI) remains a significant clinical challenge. We examined the association between the 3-week change in forced vital capacity (FVC) and time to successful decannulation, including possible nonlinear threshold effects.

**Methods:**

This prospective observational cohort study enrolled 56 tracheostomized patients with ABI. FVC was measured at baseline (T0) and at 3 weeks (T1, ΔFVC = FVC_T1 − FVC_T0) using ATS/ERS-compliant spirometry. The primary outcome was time from T0 to successful decannulation (≥72 h without reintubation or reinsertion). Multivariable linear regression, generalized additive models, and threshold effect analysis were performed after adjustment for age, sex, disease duration, diagnosis, and hemiplegic side.

**Results:**

The cohort (*N* = 56; mean age 51.0 ± 7.6 years; 55.4% male) comprised cerebral infarction (44.6%), cerebral hemorrhage (33.9%), and traumatic brain injury (21.4%). In the adjusted linear model, each 1-L increase in ΔFVC was associated with 8.7 days shorter time to decannulation (*β* = −8.677 days/L, 95% CI: −12.399 to −4.955, *p* < 0.001). A threshold effect was identified at ΔFVC = 0.52 L: below this threshold, the association was pronounced (*β* = −13.616 days/L, *p* < 0.001); above it, the association was attenuated and non-significant (*β* = −2.244 days/L, *p* = 0.492).

**Conclusion:**

Interval FVC monitoring may serve as a practical adjunct in decannulation assessment. A ΔFVC of 0.52 L or greater may serve as a clinically useful reference point for initiating comprehensive decannulation evaluation in patients with ABI undergoing pulmonary rehabilitation. This threshold should be interpreted cautiously and validated in larger multicenter studies.

## 1 Introduction

Acquired brain injury (ABI) refers to brain damage occurring after birth from traumatic or nontraumatic causes, including traumatic brain injury, stroke, and hypoxia (1, 2). As a major cause of death and disability worldwide, ABI places a growing burden on healthcare systems (3–7). In severe cases, impaired airway protection and reduced central respiratory drive often lead to respiratory failure and the need for tracheostomy (8, 9). A major challenge thereafter is determining the appropriate time for decannulation (10, 11). This decision has important clinical consequences. Premature decannulation may increase the risk of reintubation, which is associated with higher mortality, more hospital-acquired pneumonia, and longer intensive care unit (ICU) stays (12). Delayed decannulation, by contrast, prolongs tracheostomy dependence and may increase respiratory complications, length of hospitalization, and mortality. More broadly, prolonged invasive airway support has been associated with higher rates of ventilator-associated pneumonia, extended hospital stay, and increased mortality (13–15). Optimized decannulation timing is therefore essential.

Although multidisciplinary assessment is recommended (16, 17), objective physiological criteria remain limited (18). In practice, decannulation decisions still rely largely on expert opinion and clinical judgment because no universally accepted criteria or threshold values have been established (18). More objective indicators are needed to support decision-making. Current decannulation assessment generally integrates neurological recovery with respiratory status (10, 11). In patients with ABI, neurological impairment often leads to respiratory muscle weakness. Forced vital capacity (FVC), defined as the total volume of air forcibly exhaled after maximal inspiration, reflects both lung volume and respiratory muscle performance. It may therefore be a useful physiological marker of respiratory capacity and decannulation potential (19). Previous studies suggest that improvements in FVC after respiratory muscle training are associated with successful weaning and may be relevant to decannulation assessment in this population (11, 19, 20). However, the use of FVC in ABI has limitations. Patients may have impaired consciousness, cognitive deficits, or aphasia, which can reduce their ability to follow instructions and compromise measurement reliability (19, 21). In addition, little is known about whether short-term changes in FVC over a defined rehabilitation period are associated with time to successful decannulation (21). The possibility of a nonlinear relationship, including a clinically meaningful threshold, also remains unclear (20). Given these uncertainties, FVC should be interpreted within a broader framework that includes neurological status and airway management ability (22).

We hypothesized that greater improvement in FVC over the 3-week rehabilitation period (ΔFVC) would be independently associated with shorter time to successful decannulation, and that this association would exhibit a nonlinear threshold pattern, with the strongest effects observed at lower magnitudes of ΔFVC change.

To address this gap, we conducted a prospective observational study to examine the nonlinear association between the 3-week change in FVC (ΔFVC) and time to decannulation in tracheostomized patients with ABI undergoing standardized rehabilitation. We also sought to identify a potential threshold that could help inform decannulation assessment during rehabilitation.

## 2 Materials and methods

### 2.1 Study subjects

This study was conducted in the Department of Rehabilitation Medicine, Guangdong Sanjiu Brain Hospital, from January to June 2025. Newly admitted patients were screened through the hospital electronic medical record system, and eligibility was confirmed by rehabilitation physicians. The study was approved by the Ethics Committee of Guangdong Sanjiu Brain Hospital (Approval No. 202501006) and registered in the Chinese Clinical Trial Registry (Registration No. ChiCTR2500097604). Before enrollment, the study purpose, procedures, and potential risks were explained to patients and their families, and written informed consent was obtained from the patients or their legal representatives in accordance with the Declaration of Helsinki and relevant local regulations (22).

Inclusion criteria were as follows: diagnosis of ABI, including cerebral hemorrhage, cerebral infarction ischemic stroke, including large-artery atherosclerotic and cardioembolic subtypes, or traumatic brain injury, confirmed by computed tomography or magnetic resonance imaging; first-ever ABI; endotracheal intubation followed by tracheostomy within the preceding 6 months; hemodynamic stability with clear consciousness, defined as a Glasgow Coma Scale score of 13 or higher and no significant cognitive impairment as judged by a rehabilitation physician; at least moderate ability to cooperate, defined as a score of 4 or higher on the five standardized questions; age 18–80 years; and written informed consent.

Exclusion criteria were as follows: severe concomitant cardiovascular disease; structural damage to the chest wall or respiratory muscles (e.g., severe chest wall deformity, spinal deformity affecting thoracic cage integrity), paralysis of respiratory muscles, or anatomical abnormalities affecting the respiratory muscles; contraindications to respiratory muscle training, including airway bleeding, pneumothorax, hemodynamic instability, pulmonary bullae, open pulmonary tuberculosis, pleural adhesions, and pleural thickening; severe facial and tongue paralysis; and severe scoliosis and kyphosis.

### 2.2 Study design

This prospective observational cohort study examined the association between the 3-week change in FVC (ΔFVC) and time to successful decannulation in tracheostomized patients with ABI. All enrolled patients received a standardized pulmonary rehabilitation protocol to minimize confounding related to treatment intensity. The protocol consisted of four distinct components: (1) airway management and basic nursing care, (2) external diaphragm pacing therapy, (3) manual pulmonary rehabilitation, and (4) other conventional rehabilitation measures. Detailed specifications for each component are provided in section 2.6. Assessments were performed at baseline (T0), defined as the first day of structured respiratory rehabilitation, and at 3 weeks (T1). The 3-week interval was prespecified because it corresponds to the standard inpatient pulmonary rehabilitation cycle in our department and aligns with the period when formal decannulation readiness assessments are typically initiated. It was also considered sufficient to capture early respiratory changes relevant to decannulation and was informed by previous rehabilitation studies (19). Of the 60 enrolled participants, 4 withdrew during follow-up Two cases of increased cerebrospinal fluid, one case of secondary intracerebral hemorrhage, and one case of epileptic seizures during treatment., yielding a final analytic cohort of 56 participants (93.3% completion). These 4 patients were excluded from all analyses. All analyzed participants achieved successful decannulation during the study period; therefore, time to decannulation was analyzed as a continuous outcome. The enrolled cohort comprised patients with cerebral infarction, cerebral hemorrhage, and traumatic brain injury.

### 2.3 Sample size calculation

The sample size was estimated based on our previously published study protocol (23) and data from an internal pilot study (*n* = 20), which suggested an expected correlation of approximately 0.40 between ΔFVC and time to decannulation. Assuming a two-sided significance level of 0.05 and 80% power, the minimum required sample size was 47 participants. To allow for a dropout rate of up to 20%, 60 participants were enrolled. Four withdrew during follow-up, yielding a final analytic cohort of 56 participants (93.3% completion).

### 2.4 Variables and outcome measures

#### 2.4.1 Primary exposure variable

The primary exposure variable was ΔFVC, defined as the change in forced vital capacity between T0 and T1: $\text{ΔFVC}=\mathrm{FV}C_{T1}-\mathrm{FV}C_{T0.}$

#### 2.4.2 Primary outcome

The primary outcome was time to successful decannulation, defined as the number of days from T0 to the first tracheostomy tube removal that was sustained for at least 72 consecutive hours. Successful decannulation was defined as maintaining independence from the tracheostomy tube for at least 72 consecutive hours without reintubation or tracheostomy reinsertion (23, 24). Decannulation failure was defined as the occurrence, within the initial 72-h post-decannulation observation period, of any need for reintubation, reinsertion of the tracheostomy tube, invasive mechanical ventilation, or other invasive airway management due to acute upper-airway compromise, secretion retention, or severe pulmonary complications.

All decannulation decisions followed a standardized clinical protocol based on established physiological and functional criteria. Patients were considered eligible for decannulation only if they met all of the following conditions:

- 1. Stable spontaneous breathing and normothermia.
- 2. At least 48 h of substantially reduced, thin airway secretions.
- 3. Recovery of strong spontaneous cough and swallowing reflexes.
- 4. Fiberoptic laryngoscopy or three-dimensional upper-airway computed tomography reconstruction confirming no significant laryngeal or tracheal stenosis, granulation tissue, or polyps.
- 5. Chest radiography or computed tomography showing resolved or markedly improved pulmonary infection.
- 6. Fiberoptic endoscopic evaluation of swallowing (FEES) confirming swallowing safety with a Penetration Aspiration Scale (PAS) score ≤ 5 (25).
- 7. Tolerance of full tracheostomy tube capping for at least 24 h without respiratory distress or signs of worsening infection.

#### 2.4.3 Secondary outcome measures

Secondary outcomes included the Borg Scale, the Modified Barthel Index (MBI), and post—decannulation pneumonia.

The Borg Scale and MBI were assessed at T0 and T1. The Borg Scale is a validated instrument for evaluating perceived shortness of breath and respiratory symptom burden. It is scored from 0 (indicating no breathlessness) to 10 (representing maximal breathlessness). The Modified Barthel Index is a validated 10—item scale used to measure the degree of independence in activities of daily living. Scores on this scale range from 0 to 100, with higher scores denoting greater functional independence.

Post—decannulation pneumonia was monitored between the end of the initial 72—h observation period and T1.

In addition, several respiratory and diaphragmatic variables were measured at T0 and T1 to characterize changes in respiratory muscle performance and diaphragmatic function during rehabilitation. These variables included maximal inspiratory pressure (MIP), maximal expiratory pressure (MEP), diaphragmatic excursion (DE), diaphragm thickness at end—inspiration (IDT), diaphragm thickness at end—expiration (EDT), and diaphragmatic thickening fraction (DTF).

#### 2.4.4 Covariates

Baseline demographic and clinical covariates included age, sex, disease duration, primary diagnosis, and hemiplegic side. Disease duration was defined as the time from ABI onset to T0. Primary diagnosis was categorized as cerebral hemorrhage, cerebral infarction, or traumatic brain injury. Hemiplegic side was classified as left or right.

### 2.5 Assessment procedures for pulmonary and functional variables

To minimize bias, clinicians responsible for decannulation decisions were blinded to real-time pulmonary function results. Pulmonary function assessors were independent and were not involved in decannulation decisions or rehabilitation delivery. Pulmonary and functional variables were assessed at T0 and T1 by designated pulmonary rehabilitation personnel using a standardized operating procedure. All assessments were performed in the Department of Rehabilitation Medicine, not in the ICU. Patients had been medically stabilized and transferred from ICU prior to enrollment.

#### 2.5.1 FVC measurement

Forced vital capacity (FVC) was measured at T0 and T1 using the X1 Portable Spirometer (Saike Medical Equipment Co., Ltd., Xiamen, China), which complies with relevant American Thoracic Society/European Respiratory Society standards (26) and has been widely used in clinical settings in China (19).

FVC was selected over FEV1 as the primary pulmonary function measure because it is more reliably performed by tracheostomized patients with acquired brain injury, who often have variable cooperation; FVC is less sensitive to early termination artifacts that are common in this population, and it provides a comprehensive index of ventilatory capacity that is directly relevant to decannulation readiness.

The measurement protocol was adapted for tracheostomized patients. Before testing, cuff pressure was adjusted to 25–30 cmH₂O, and airway secretions were suctioned to ensure a patent airway. The spirometer was connected to the tracheostomy tube using a dedicated adapter. Patients were positioned upright or semi—recumbent. Trained assessors provided standardized instructions and encouragement throughout the procedure. After several tidal breaths, patients were guided to exhale completely from total lung capacity (TLC) to residual volume (RV), followed immediately by a maximal forced inspiration and expiration to obtain FVC. Each parameter was measured three times consecutively, with at least 1 min of rest between attempts to prevent fatigue. Three acceptable maneuvers were obtained, and the highest FVC value was recorded. To ensure data quality, reproducibility required a difference of no more than 150 mL between the two best maneuvers (27).

#### 2.5.2 MIP and MEP measurement

Maximal inspiratory pressure (MIP) and maximal expiratory pressure (MEP) were measured using the same portable spirometer under standardized conditions. MIP and MEP measurements were performed at T0 and T1. All measurements were performed after the patients were deemed hemodynamically stable and able to cooperate with testing. Before measurement, tracheostomy tube cuff pressure was adjusted to 25–30 cmH₂O and airway secretions were thoroughly suctioned. Measurements were performed with patients in a semi-recumbent position, and the spirometer was connected to the tracheostomy tube through a disposable bacterial filter. For MIP assessment, patients were instructed to exhale completely to residual volume before performing a sustained maximal inspiratory effort. For MEP assessment, patients were instructed to inhale deeply to total lung capacity before performing a sustained maximal expiratory effort. Each maneuver was performed three times with at least 1 min of rest between attempts, and the highest value was used for analysis (19).

#### 2.5.3 Diaphragmatic ultrasound evaluation

Diaphragmatic ultrasound was performed using a portable color Doppler ultrasound system (model SII, Sonos Medical Devices Co., Ltd.) (27). All measurements were obtained with patients in the supine position and the head of the bed elevated to 0°–30°. Right hemidiaphragm thickness was measured at the zone of apposition between the anterior and midaxillary lines in the eighth to tenth intercostal spaces using a high-frequency linear-array probe. End-expiratory diaphragm thickness (EDT) and end-inspiratory diaphragm thickness (IDT) were recorded at the corresponding respiratory phases, and diaphragmatic thickening fraction (DTF) was calculated as: $\mathrm{DTF}\%=\frac{\mathrm{IDT}-\mathrm{EDT}}{\mathrm{EDT}}\times 100\%.$ Diaphragmatic excursion (DE) was measured using a phased-array or convex-array probe placed at the right subcostal margin along the midclavicular or anterior axillary line. M-mode imaging was acquired through the liver acoustic window, with at least three quiet respiratory cycles recorded for each patient. DE was measured as the vertical distance between the end-inspiratory peak and end-expiratory trough for each cycle, and the average value was used for analysis (28).

#### 2.5.4 Assessment of the Borg Scale and Modified Barthel Index

The Borg Scale was administered by trained assessors at T0 and T1 to evaluate perceived dyspnea and respiratory symptom burden. Patients were seated comfortably and asked to rate their breathlessness on the 0–10 category scale after a brief 5-min rest. The scale was presented both visually (on a card) and read aloud by the assessor using standardized instructions. Anchor descriptors were provided at each integer point (0, “no breathlessness at all,” 10, “maximal breathlessness”). The score was recorded immediately after the patient’s response. The Modified Barthel Index (MBI) was administered by trained rehabilitation therapists at T0 and T1 to assess functional independence in activities of daily living. The MBI consists of 10 items covering personal care (bowel control, bladder control, grooming, toilet use, feeding) and mobility (transfer, mobility, stairs, dressing, bathing). Each item is scored on an ordinal scale (0–1, 0–2, or 0–3 depending on the item), with total scores ranging from 0 to 100. Higher scores indicate greater functional independence. All items were assessed through structured observation and interview with the patient, and each item was scored according to the standard MBI criteria.

### 2.6 Rehabilitation treatment and decannulation protocol

All enrolled patients received a standardized pulmonary rehabilitation protocol to minimize confounding related to treatment intensity. The protocol included the following components: All enrolled patients received a standardized pulmonary rehabilitation protocol to minimize confounding related to treatment intensity. The protocol consisted of four distinct components: (1) airway management and basic nursing care, (2) external diaphragm pacing therapy, (3) manual pulmonary rehabilitation, and (4) other conventional rehabilitation measures. Detailed specifications for each component are provided.

(1) Airway management and basic nursing measures.

Airway humidification: The humidification fluid temperature was maintained at 37.0 °C with 100% relative humidity. The humidification level was adjusted based on the patient’s clinical status to prevent over—or under—humidification. Sputum characteristics and physical examination findings were monitored to assess efficacy.

Balloon management: A handheld balloon pressure gauge was used to maintain cuff pressure between 25 and 30 cmH₂O. Pressure was measured every 6–8 h, and fluid in the pilot balloon tubing was removed. During measurement, the recorded pressure was targeted to be approximately 2 cmH₂O above the set value. Secretions adhering to the cuff were cleared every 2–3 h.

Respiratory secretion management: Endotracheal suctioning was performed as clinically indicated, typically using a negative pressure of 80–120 mmHg. For highly viscous secretions, pressure could be increased to a maximum of 150 mmHg. Each suctioning episode did not exceed 15 s, with no more than two consecutive attempts.

Oral care: Oral hygiene was performed at least twice daily using a designated oral care solution.

Wound management: The tracheostomy site was kept clean and dry, using specialized tracheostomy gauze or foam dressings. The sterile gauze tracheostomy tube dressing was changed twice daily.

(2) External diaphragm pacing therapy

External diaphragm pacing was performed using a dedicated EDP device. Two self-adhesive surface electrodes were placed bilaterally: one over the phrenic nerve roots at C3–C5 (anterior neck) and the other over the 7th–9th intercostal spaces at the midclavicular line. Electrode placement was confirmed by observing visible diaphragmatic contraction during test stimulation. Stimulation parameters: frequency 1–2 Hz, pulse width 200–300 μs, current intensity 15–30 mA, duration 30–60 min/session. Monitoring included SpO₂, heart rate, and respiratory rate. Contraindications (pneumothorax, active pulmonary tuberculosis, implanted cardiac pacemaker) were assessed before each session.

(3) Pulmonary rehabilitation manual therapy. To ensure consistency, all manual therapy was performed by the same certified pulmonary rehabilitation therapist. The therapy included the following elements:

Respiratory muscle relaxation: With the patient supine, the therapist sequentially relaxed the intercostal muscles along the costal margins. Downward or downward—inward traction was applied to the thoracic cage during expiration to assist exhalation.

Airway clearance: This combined postural drainage, percussive tapping, and vibration. Sessions (30 min each) were conducted with the patient in sitting, supine, lateral, or prone positions, five times per week for 3 weeks.

Respiratory training: A threshold load device was used, with an initial intensity set at 30% of the patient’s maximal inspiratory pressure (MIP). Patients, seated or standing, performed rapid deep inspirations to total lung capacity, followed by a slow 6-s exhalation. Each set comprised 10 repetitions; three sets were performed per session, with 2-min rest intervals between sets. This was supplemented with pursed—lip and diaphragmatic breathing. Training intensity was increased weekly by 5–10% of MIP, not exceeding 60% of MIP. Sessions lasted 20 min daily, 5 days per week, for 4 weeks.

(4) Other conventional rehabilitation measures included postural management (head elevation ≥30°, turning every 2 h, proper limb positioning), swallowing training (oral sensorimotor training, lip movement exercises, Mendelsohn maneuver; 30 min/session, 5 times/week for 3 weeks), and exercise therapy (passive/active range of motion for upper and lower limbs, turning, transfer, and sitting training; 15–20 min/day). Auxiliary modalities such as tilt tables, cycle ergometers, acupuncture, and transcranial magnetic stimulation (TMS) were integrated based on individual clinical needs.

### 2.7 Follow-up

From T0 onward, decannulation status was assessed and recorded daily. After each decannulation attempt, patients entered an initial 72-h observation period. Any need for reintubation, tracheostomy reinsertion, or other invasive airway management during this period was classified as decannulation failure. Patients who remained free of these events for 72 h were considered successfully decannulated. Follow-up for the primary outcome continued from T0 until successful decannulation or T1 if decannulation was not achieved earlier. Patients who achieved successful decannulation before T1 entered the 72-h observation period and were then monitored until T1 for post-decannulation complications. Of the 56 analyzed patients, 55 achieved decannulation before T1 and 1 reached decannulation at T1. All patients who decannulated before T1 were successfully decannulated (no decannulation failures) and completed the 72-h observation period. After successful decannulation was confirmed, patients were monitored until T1 for post-decannulation complications such as pneumonia. Post-decannulation pneumonia was classified as a complication and managed according to standard clinical protocols, distinct from decannulation failure, which was defined as the need for reintubation, reinsertion, or invasive ventilation within the 72-h observation window. Events occurring after the initial 72-h observation period were classified as post-decannulation complications rather than decannulation failure.

### 2.8 Statistical analysis

Descriptive statistics were reported as mean ± standard deviation (SD) for normally distributed continuous variables and median [interquartile range (IQR)] for non-normally distributed continuous variables, based on the Shapiro–Wilk test. Categorical variables were expressed as counts and percentages.

Univariate linear regression analyses were performed to examine associations between time to decannulation and age, sex, disease duration, primary diagnosis, hemiplegic side, and ΔFVC. Multivariable linear regression was then used to estimate the independent association between ΔFVC and time to decannulation after adjustment for age, sex, disease duration, primary diagnosis, and hemiplegic side.

Because a nonlinear association was suspected, the dose–response relationship was further explored using generalized additive models (GAMs) with penalized spline smoothing after adjustment for diagnosis, hemiplegic side, age, sex, and disease duration. When a threshold pattern was identified, a two-piece linear regression model was fitted to estimate the inflection point (K) for ΔFVC. Differences in slopes between the two segments were assessed, and model fit was compared with that of the simple linear model using an F test.

All analyses were conducted in R software (version 4.3.2; R Foundation for Statistical Computing), primarily with the mgcv and rms packages. A two-sided *p* < 0.05 was considered statistically significant.

## 3 Results

### 3.1 Baseline characteristics of study participants

The study included a total of 56 patients. The mean age of the cohort was 51.00 ± 7.57 years, and 55.4% were male. The detailed baseline characteristics are presented in Table 1. At T0, participants showed impaired respiratory function (FVC: 1.76 ± 0.16 L; MIP: 23.57 ± 3.46 cmH₂O; MEP: 26.57 ± 4.37 cmH₂O; Borg Scale: 7.02 ± 0.81) and limited functional independence (MBI: 50.02 ± 5.06). By T1, FVC increased to 2.22 ± 0.27 L (*Δ* = 0.46 ± 0.17 L, +26.1%), respiratory muscle strength improved (MIP: 33.07 ± 3.95 cmH₂O, Δ = +9.48 ± 2.52 cmH₂O; MEP: 35.80 ± 4.58 cmH₂O, Δ = +9.07 ± 3.25 cmH₂O), diaphragmatic function improved (DE: 3.45 ± 0.56 cm, IDT: 30.27 ± 5.29 mm, DTF: 31.95 ± 4.70%), functional independence increased (MBI: 72.79 ± 8.49), and dyspnea perception decreased (Borg Scale: 3.29 ± 0.92) (Table 2). During follow-up, post-decannulation pneumonia occurred in 4 patients (7.1%), and no other major respiratory complications were observed. These 4 patients received standard-of-care antibiotic therapy; none required reintubation, tracheostomy reinsertion, or invasive mechanical ventilation during the 72-h post-decannulation observation period or during continued monitoring until T1 (Table 3).

**Table 1:** Baseline characteristics of the study participants (*N* = 56).

Variable	Category	Value
Age (years)	–	51.00 ± 7.57(33–60)/51
Disease duration (weeks)	–	6.38 ± 2.03(2–12)/6
Sex	Male	31 (53.3%)
	Female	25 (46.7%)
Primary diagnosis	Cerebral hemorrhage	19 (35.0%)
	Cerebral infarction	25 (41.7%)
	Traumatic brain injury	12 (23.3%)
Hemiplegic side	Left	30 (53.6%)
	Right	26 (46.4%)

Continuous variables are presented as mean ± SD (min–max)/median. Categorical variables are presented as n (%). SD, standard deviation; min, minimum; max, maximum; N, number of participants. FVC, forced vital capacity.

**Table 2:** Respiratory function and clinical measures at baseline and 3 weeks.

Variable	Baseline (T0)	3 weeks (T1)	Change (T1 − T0)
FVC (L)	1.76 ± 0.16	2.22 ± 0.27	0.46 ± 0.17
MIP (cmH₂O)	23.57 ± 3.46	33.07 ± 3.95	9.48 ± 2.52
MEP (cmH₂O)	26.57 ± 4.37	35.80 ± 4.58	9.07 ± 3.25
DE (cm)	2.67 ± 0.36	3.45 ± 0.56	–
IDT (mm)	24.72 ± 3.65	30.27 ± 5.29	–
EDT (mm)	20.43 ± 2.76	22.88 ± 3.57	–
DTF (%)	20.80 ± 2.59	31.95 ± 4.70	–
MBI	50.02 ± 5.06	72.79 ± 8.49	–
Borg Scale	7.02 ± 0.81	3.29 ± 0.92	–

Continuous variables are presented as mean ± SD where appropriate. SD, standard deviation; T0, baseline assessment; T1,post-intervention assessment; FVC, forced vital capacity; ΔFVC, absolute change in FVC; MIP, maximal inspiratory pressure; MEP, maximal expiratory pressure; DE, diaphragmatic excursion; IDT, end-inspiratory diaphragm thickness; EDT, end-expiratory diaphragm thickness; DTF, diaphragmatic thickening fraction; MBI, Modified Barthel Index.

**Table 3:** Decannulation outcomes and post-decannulation complications.

Outcome measure	Category	Value
Time to decannulation (days)	–	10.58 ± 2.93(5–18)/10
Post-decannulation pneumonia	No	52 (92.9%)
	Yes	4 (7.1%)

Continuous variables are presented as mean ± SD (min–max)/median. Categorical variables are presented as n (%).pneumonia cases were treated with antibiotics and did not meet criteria for decannulation failure.

### 3.2 Univariate regression analysis

Univariate linear regression results are presented in Table 4. Greater ΔFVC was significantly associated with a shorter time to decannulation (*β* = −10.524, 95% CI: −14.253 to −6.796, *p* < 0.001), whereas older age was associated with a longer time to decannulation (*β* = 0.248, 95% CI: 0.165–0.332, *p* < 0.001). Primary diagnosis was also associated with time to decannulation. Compared with cerebral hemorrhage, traumatic brain injury was associated with shorter time to decannulation, whereas cerebral infarction (ischemic stroke) was not. Disease duration, hemiplegic side, and sex were not significantly associated with time to decannulation.

**Table 4:** Univariate linear regression analysis of factors associated with time to decannulation.

Variable	β (95% CI)	*P*
ΔFVC (L)	−10.524 (−14.253 to −6.796)	<0.001
Age (years)	0.248 (0.165–0.332)	<0.001
Diagnosis (reference: cerebral hemorrhage)		
Traumatic	−3.842 (−5.817 to −1.867)	<0.001
Cerebral infarction	−1.002 (−2.632 to 0.628)	0.223
Disease duration (weeks)	0.285 (−0.102 to 0.672)	0.146
Hemiplegic side (reference: right)		
Left	−0.205 (−1.822 to 1.412)	0.800
Sex (reference: female)		
Male	−0.052 (−1.675 to 1.571)	0.949

Values are presented as β (95% CI) and *p*-value. Statistical significance was set at *p* < 0.05. Variables with *p* < 0.05 were considered statistically significant. Reference categories are indicated for categorical variables. β, regression coefficient; CI, confidence interval; ΔFVC, absolute change in forced vital capacity from baseline (T0) to post-intervention (T1); T0, baseline assessment; T1, post-intervention assessment; SD, standard deviation.

### 3.3 Multivariable regression analysis

Multivariable linear regression was used to assess whether the association between ΔFVC and time to decannulation was independent of potential confounders, with adjustment for prespecified clinically relevant covariates including age, disease duration, primary diagnosis, hemiplegic side, and sex (Table 5). In the unadjusted model, each 1-L increase in ΔFVC was associated with a 10.5-day shorter time to decannulation (*β* = −10.524 days/L, 95% CI: −14.253 to −6.796, *p* < 0.001). After adjustment, the association remained significant, with each 1-L increase in ΔFVC corresponding to an 8.7-day shorter time to decannulation (*β* = −8.677 days/L, 95% CI: −12.399 to −4.955, *p* < 0.001).

**Table 5:** Multivariable linear regression analysis of the association between ΔFVC and time to decannulation.

Variable	Unadjusted model		Adjusted model	
	β (95% CI)	*P*	β (95% CI)	*P*
ΔFVC (L)	−10.524 (−14.253 to −6.796)	<0.001	−8.677 (−12.399 to −4.955)	<0.001

The adjusted model accounted for age, disease duration, primary diagnosis, hemiplegic side, and sex. β, regression coefficient; CI, confidence interval; ΔFVC, absolute change in forced vital capacity from baseline (T0) to post-intervention (T1); T0, baseline assessment; T1, post-intervention assessment; SD, standard deviation.

### 3.4 Smooth curve fitting and threshold effect analysis

#### 3.4.1 Smooth curve fitting

**Figure 1:**
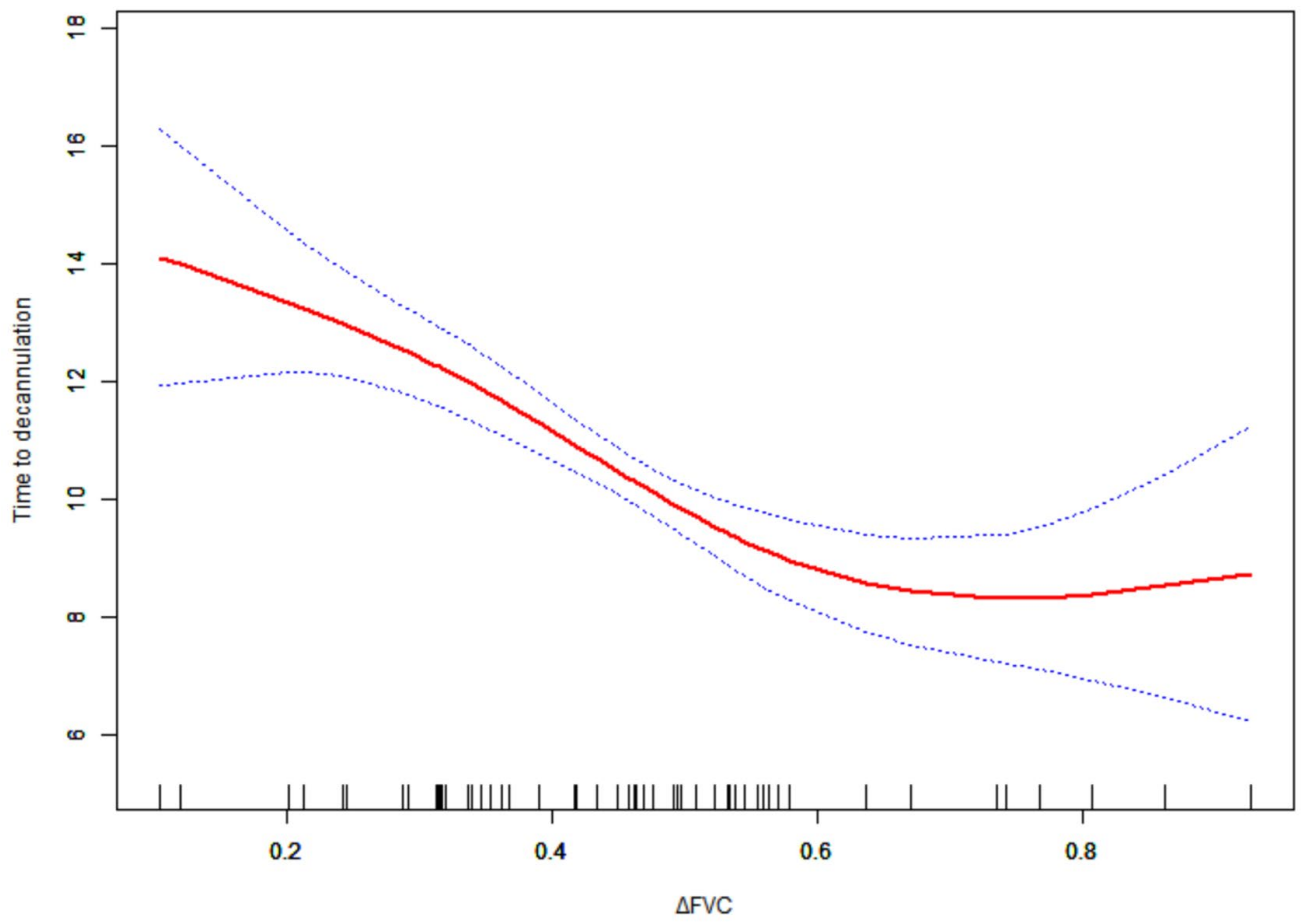
Relationship between ΔFVC and time to decannulation. The smooth curve, generated using a generalized additive model (GAM), illustrates the relationship between ΔFVC and time to decannulation. The solid red line shows the fitted relationship, and the blue dashed area represents the 95% confidence interval. The x-axis indicates ΔFVC (L) and the y-axis indicates time to decannulation (days). The model was adjusted for diagnosis, hemiplegic side, age, sex, and time since brain injury onset.

To further characterize the association between ΔFVC and time to decannulation, a generalized additive model was fitted after adjustment for diagnosis, hemiplegic side, age, sex, and disease duration. As shown in Figure 1, the association was nonlinear. Larger increases in ΔFVC were generally associated with shorter time to decannulation, but the rate of reduction diminished at higher ΔFVC values, suggesting a plateau effect. The apparent transition point was approximately ΔFVC = 0.52 L.

Threshold effect analysis was then performed to further define the nonlinear association between ΔFVC and time to decannulation (Table 6). In the conventional linear model, ΔFVC was significantly and inversely associated with time to decannulation. A two-piece linear regression model identified an inflection point at ΔFVC = 0.52 L, and model comparison supported the segmented model over the simple linear model (*p* = 0.022).

**Table 6:** Threshold effect analysis of the relationship between ΔFVC and time to decannulation.

Category.	Metric	Estimate	*P*
Model I	Linear	−8.677 (−12.399, −4.955)	**<0.001**
Model II			
	Two-piece linear, Threshold (K)	0.52	–
	Effect 1 (ΔFVC< K)	−13.616 (−19.110, −8.123)	**<0.001**
	Effect 2 (ΔFVC> K)	−2.244 (−8.755, 4.267)	0.492
	Difference (Effect 2 − Effect 1)	11.372 (1.732, 21.013)	**0.022**
Model comparison	F test (df = 1)	*F* = 5.632	**0.022**
Sample distribution	Sample size ≤ K	36	–
Sample distribution	Sample size > K	20	–

Values are presented as estimate, β (95% CI), F, and *p*-value as appropriate. Statistical significance (*p* < 0.05). *p*-values meeting this threshold are displayed in bold. Threshold (piecewise linear) regression analysis was used to identify the optimal ΔFVC threshold (K) for decannulation time. All models were adjusted for diagnosis, hemiplegic side, age, sex, and disease duration (weeks). The outcome variable was time to decannulation (days); the exposure variable was ΔFVC (L). β indicates the change in decannulation time per 1-L increase in ΔFVC. β, regression coefficient; CI, confidence interval; ΔFVC, absolute change in forced vital capacity from baseline (T0) to post-intervention (T1); T0, baseline assessment; T1, post-intervention assessment; K, threshold value (optimal cutoff); F, F-statistic; df, degrees of freedom; SD, standard deviation; n, number of participants.

Below the threshold, each 1-L increase in ΔFVC was associated with a 13.616-day shorter time to decannulation (95% CI, −19.110 to −8.123, *p* < 0.001). Above the threshold, the association was attenuated and no longer statistically significant (*β* = −2.244 days/L, 95% CI: −8.755 to 4.267, *p* = 0.492). These findings suggest that the inverse association between ΔFVC and time to decannulation was stronger when ΔFVC was below 0.52 L, whereas further increases beyond this level were associated with limited additional reduction in decannulation time.

### 3.5 GAM smooth curve analysis (ALL)

To comprehensively assess the associations between all respiratory parameters and time to decannulation, generalized additive model (GAM) smooth curve fitting was performed for each of the 19 prespecified exposure variables (Supplementary Table S1), adjusting for age, sex, diagnosis, disease duration, and hemiplegic side. Threshold piecewise regression was applied to all prespecified variables.

We systematically examined all 19 respiratory parameters using two complementary statistical approaches. Both GAM smooth curve fitting and threshold piecewise regression consistently identified ΔFVC as the only variable with robust nonlinear and threshold effects $\mathrm{Adj}.R^{2}=0.59,K=0.52L,p=0.022$ . Importantly, DE_T1 $p=0.0005,\mathrm{Adj}.R^{2}=0.49$ and DTF_T1 $p=0.0064,\mathrm{Adj}.R^{2}=0.43$ also showed significant nonlinear associations—confirming that diaphragmatic function is clinically relevant to decannulation timing. All other individual parameters showed no significant nonlinear or threshold effects.

## 4 Discussion

In this prospective study of tracheostomized patients with ABI undergoing pulmonary rehabilitation, greater improvement in FVC over 3 weeks was independently associated with shorter time to decannulation. We also identified a nonlinear association, with a threshold at ΔFVC = 0.52 L. Below this value, greater increases in ΔFVC were associated with a more pronounced reduction in time to decannulation, whereas above it the association weakened.

These findings are broadly consistent with previous evidence supporting FVC as a clinically relevant parameter in decannulation assessment. Geiseler and Westhoff (26) reported the utility of interval FVC monitoring but did not define a threshold. Pan et al. (29) identified significant associations between spirometric parameters and decannulation outcomes, with FEV_1_ showing strong predictive performance at a cutoff of 0.42 L. Kraghede et al. (30) demonstrated that FVC measured immediately after tracheostomy sealing was significantly higher than with open tracheostomy (median 1,260 vs. 833 mL, *p* < 0.001), providing physiological evidence that FVC improves acutely once airway patency is restored. However, this acute feasibility study involved only a single post-decannulation measurement and did not capture the trajectory of pulmonary recovery. Kraghede et al. (31) subsequently reported serial daily spirometry (FVC, FEV₁, PEF) in 21 patients throughout the post-decannulation wound-healing period (up to 7 days), demonstrating that FVC improvements were significant immediately after sealing and sustained across serial daily measurements, and Soroksky et al. (32) reported improved prediction when FVC was combined with breath-hold duration. Collectively, these studies suggest that better pulmonary reserve and stronger respiratory function are associated with higher decannulation success or shorter decannulation time. Our study extends this literature by focusing on interval change in FVC rather than a single baseline value and by identifying a potential ΔFVC threshold linked to clinically meaningful shortening of decannulation time.

Differences between our threshold and prior reports may reflect differences in study population, outcome definition, and measurement strategy. These methodological variations may influence both effect size and cutoff location; therefore, direct comparisons should be made cautiously. Lower FVC has also been associated with greater neurological burden (30–33). More relevant to our findings, improvement in ΔFVC has been linked to earlier decannulation (34), whereas deterioration in ΔFVC may indicate reduced respiratory reserve after withdrawal of airway support (35). In addition, evidence that earlier decannulation may reduce the risk of diaphragmatic atrophy (36, 37) indirectly supports the value of dynamic respiratory monitoring. To our knowledge, however, no previous study has directly evaluated a nonlinear ΔFVC threshold for decannulation timing in patients with ABI.

The observed pattern may reflect a threshold-like phase of functional recovery in which gains in respiratory capacity translate more efficiently into earlier decannulation. Mechanistically, the rapid improvement below the threshold may reflect better diaphragmatic coordination, improved pulmonary compliance, and more effective cough generation, all of which are important for secretion clearance and decannulation readiness (35, 36). Once ΔFVC reaches 0.52 L or higher, additional gains in FVC may offer less incremental benefit, and other factors—such as swallowing-respiratory coordination, airway protection, and laryngeal sensory function—may become relatively more important. This interpretation is consistent with studies of extubation outcomes suggesting that coordinated swallowing and protective cough, rather than respiratory muscle strength alone, may become the key determinants of airway safety once basic respiratory reserve has been achieved (36).

In addition to ΔFVC, post-treatment diaphragmatic excursion (DE_T1, *p* = 0.0005, Adj. *R*^2^ = 0.49) and post-treatment diaphragmatic thickening fraction (DTF_T1, *p* = 0.0064, Adj. *R*^2^ = 0.43) also showed significant nonlinear associations with decannulation time, confirming that improved diaphragmatic function is clinically relevant to decannulation timing. However, neither DE_T1 nor DTF_T1 demonstrated a threshold effect, suggesting that while diaphragmatic excursion and contractile force are consistently associated with better outcomes, there is no discrete cut-off level beyond which decannulation is categorically faster—consistent with the multifactorial nature of decannulation readiness. Notably, an opposite pattern was observed between ventilatory and diaphragmatic parameters: ΔFVC (Adj. *R*^2^ = 0.59) outperformed FVC_T1 (Adj. *R*^2^ = 0.51), whereas DE_T1 (Adj. *R*^2^ = 0.49) and DTF_T1 (Adj. *R*^2^ = 0.43) substantially outperformed their change values ΔDE (Adj. *R*^2^ = 0.36) and ΔDTF (Adj. *R*^2^ = 0.15), respectively. This differential pattern may reflect underlying physiology: FVC as a composite integrative measure captures overall ventilatory reserve achieved after rehabilitation, making the magnitude of change more discriminative; in contrast, diaphragmatic excursion and thickening at T1 represent the absolute functional capacity achieved at the moment of assessment—the level that matters for airway protection—while the magnitude of prior improvement is less predictive when patients start from different baseline levels. This is further supported by the uniform absence of significant associations for all baseline (T0) variables (all *p* > 0.30, Adj. *R*^2^ ≈ 0.34), and by the consistent lack of predictive value for static anatomical measures of diaphragm thickness (IDT, EDT) at either time point, indicating that dynamic function outweighs static anatomy in determining effective airway protection.

This threshold may also have practical implications. When ΔFVC is below 0.52 L, continued emphasis on respiratory training may be especially important. When ΔFVC reaches or exceeds 0.52 L, clinical focus may need to shift toward more comprehensive assessment of swallowing, secretion management, and airway protection. In our analyses, ΔFVC showed a more consistent association with time to decannulation than isolated respiratory muscle strength or diaphragmatic variables, suggesting that in patients with ABI, FVC may serve as a more integrated marker of the overall respiratory reserve required for successful decannulation.

This study has several strengths. First, its prospective design enabled interval assessment of respiratory function during a standardized rehabilitation period. Second, FVC was measured using ATS/ERS-compliant spirometry, which improved reliability. Third, we evaluated ΔFVC as a dynamic marker rather than relying only on a single baseline value. Fourth, generalized additive modeling allowed us to detect a nonlinear association and identify a clinically interpretable threshold. Finally, the study focused on a clinically relevant population of tracheostomized patients with ABI undergoing inpatient rehabilitation, in whom objective decannulation markers remain limited.

Several limitations should also be acknowledged. First, this was a single-center observational study with a relatively small sample, which may limit generalizability. Second, only two FVC measurements were obtained during the study period. Serial measurements with shorter intervals could better characterize the trajectory of respiratory recovery and identify optimal decannulation timing; future studies should consider more frequent assessments to validate and refine the ΔFVC threshold identified in this study. Third, the cohort included different ABI etiologies, and the identified threshold may not apply equally across all subgroups. Fourth, the threshold value of 0.52 L was derived internally and has not been externally validated. Fifth, swallowing function and protective cough efficacy were not assessed as quantitative variables. All patients were required to pass a formal swallowing safety assessment (FEES with PAS score ≤ 5) and demonstrate recovery of spontaneous cough and swallowing reflexes before decannulation, as specified in the standardized protocol (section 2.4.2); however, the absence of quantitative swallowing and cough efficacy data limits our ability to quantify their independent contribution to decannulation timing. Future studies should incorporate validated quantitative measures of swallowing function (e.g., videofluoroscopic swallowing study) and cough efficacy (e.g., peak cough flow) to provide a more comprehensive assessment of decannulation readiness. In addition, because the primary analysis included only patients who achieved decannulation within the prespecified follow-up window, selection bias is possible. Finally, because the analysis focused on observed decannulation times during the study period, the findings should not be interpreted as establishing causality or replacing comprehensive clinical assessment.

## 5 Conclusion

In tracheostomized patients with ABI undergoing pulmonary rehabilitation, greater improvement in FVC over 3 weeks was associated with shorter time to decannulation. The association was nonlinear, with a potential threshold at ΔFVC = 0.52 L. interval FVC monitoring may therefore provide a practical reference for decannulation assessment during ABI rehabilitation. However, this threshold should be used as an adjunct to, rather than a substitute for, comprehensive clinical evaluation and requires validation in larger multicenter studies.

## Data Availability

The raw data supporting the conclusions of this article will be made available by the authors, without undue reservation.
