# Supplementary material for: Decannulation criteria in patients with acquired brain injury based on interval forced vital capacity monitoring: a prospective observational study
Source: Front Neurol. 2026 Jul 9;17:1798877. doi: 10.3389/fneur.2026.1798877 (PMC13391517; doi:10.3389/fneur.2026.1798877)
Supplement: Supplementary file 1 [file Data_Sheet_1.pdf]

Supplementary Material for:Decannulation criteria in patients with acquired brain injury based on interval forced vital capacity monitoring: a prospective observational study

Table S1. GAM Smooth Curve Analysis: Nonlinear Associations Between All Respiratory Parameters and Time to Decannulation

Variable	Time Point	edf	P - value	Adj. R^2	Nonlinear	Threshold
FVC (L)	T0	1.00	0.9047	0.3354	No	—
DE (cm)	T0	1.00	0.9779	0.3352	No	—
MIP (cmH_2O)	T0	1.00	0.3687	0.3462	No	—
MEP (cmH_2O)	T0	1.00	0.8539	0.3357	No	—
IDT (mm)	T0	1.00	0.8350	0.3358	No	—
EDT (mm)	T0	1.00	0.9559	0.3353	No	—
DTF (%)	T0	1.09	0.4721	0.3456	No	—
FVC (L)	T1	1.00	< 0.0001	0.5138	Yes	No
DE (cm)	T1	1.00	0.0005	0.4887	Yes	No
MIP (cmH_2O)	T1	1.00	0.0570	0.3861	Borderline	—
MEP (cmH_2O)	T1	1.00	0.0428	0.3923	Borderline	—
IDT (mm)	T1	1.00	0.9998	0.3375	No	—
EDT (mm)	T1	1.00	0.4179	0.3466	No	—
DTF (%)	T1	1.00	0.0064	0.4335	Yes	No
ΔFVC (L)	Δ	2.52	< 0.0001	0.5853	Yes	0.022
ΔDE (cm)	Δ	≈ 1	0.8012	0.3561	No	No
ΔMIP (cmH_2O)	Δ	1.10	0.1958	0.3652	No	—
ΔMEP (cmH_2O)	Δ	2.04	0.0736	0.4187	Borderline	—
ΔDTF (%)	Δ	≈ 1	0.6744	0.1531	No	No

Generalized additive model (GAM) smooth curve fitting was performed for each exposure variable to test for nonlinear associations with decannulation time, adjusting

for age, sex, diagnosis, disease duration, and hemiplegic side. All 19 variables were analyzed, N = 56 valid cases).FVC, Forced Vital Capacity； DE, Diaphragmatic Excursion；MIP, Maximal Inspiratory Pressure；MEP, Maximal Expiratory Pressure；IDT, End - Inspiratory Diaphragm Thickness； EDT, End - Expiratory Diaphragm Thickness； DTF, Diaphragmatic Thickening Fraction； Δ (prefix), change； edf, Effective Degrees of Freedom； Adj. R^2, Adjusted R - squared； T0, Baseline Assessment；T1, Post - Intervention Assessment；
